# Enhancing the Liquid-Phase Exfoliation of Graphene in Organic Solvents upon Addition of *n*-Octylbenzene

**DOI:** 10.1038/srep16684

**Published:** 2015-11-17

**Authors:** Sébastien Haar, Mirella El Gemayel, Yuyoung Shin, Georgian Melinte, Marco A. Squillaci, Ovidiu Ersen, Cinzia Casiraghi, Artur Ciesielski, Paolo Samorì

**Affiliations:** 1 ISIS & icFRC, Université de Strasbourg & CNRS, 8 allée Gaspard Monge, 67000 Strasbourg, France; 2 School of Chemistry, Oxford Road, University of Manchester, Manchester M13 9PL, United Kingdom; 3 Institut de Physique et Chimie des Matériaux de Strasbourg (IPCMS), UMR 7504, 23 rue du Loess, 67037 Cédex 08 Strasbourg, France

## Abstract

Due to a unique combination of electrical and thermal conductivity, mechanical stiffness, strength and elasticity, graphene became a rising star on the horizon of materials science. This two-dimensional material has found applications in many areas of science ranging from electronics to composites. Making use of different approaches, unfunctionalized and non-oxidized graphene sheets can be produced; among them an inexpensive and scalable method based on liquid-phase exfoliation of graphite (LPE) holds potential for applications in opto-electronics and nanocomposites. Here we have used *n*-octylbenzene molecules as graphene dispersion-stabilizing agents during the graphite LPE process. We have demonstrated that by tuning the ratio between organic solvents such as N-methyl-2-pyrrolidinone or *ortho*-dichlorobenzene, and *n*-octylbenzene molecules, the concentration of exfoliated graphene can be enhanced by 230% as a result of the high affinity of the latter molecules for the basal plane of graphene. The LPE processed graphene dispersions were further deposited onto solid substrates by exploiting a new deposition technique called spin-controlled drop casting, which was shown to produce uniform highly conductive and transparent graphene films.

Graphene, a 2D honeycomb lattice of carbon atoms, has been the subject of countless studies since its first experimental isolation by mechanical exfoliation in 2004^1^,^2^. Its extraordinary electrical, thermal, mechanical and optical properties qualified it as a material of choice for applications in electronics^3^,^4^, photonics^5^, composites including paints and coating^6^,^7^,^8^, energy generation and storage^9^,^10^,^11^, sensors^12^,^13^ and bio-applications^14^. The technological exploitation of graphene’s superior properties triggered the search for up-scalable manufacturing process. However, one of the critical factors that limit the use of some fabrication processes already available is the production of size-controlled defect-free graphene sheets. Because of this reason, a great effort has been devoted to the to production of both single- and few-layer graphene sheets by using more than twenty different approaches^15^,^16^,^17^. These methods can be classified into two distinct strategies, i.e. the *bottom-up* and the *top-down*. The former relies on the formation of covalent bonds between atoms or suitably designed small molecular building blocks to form 2D network by using chemical^18^ or physical processes, like chemical vapor deposition (CVD)^19^,^20^, molecular beam epitaxy^21^,^22^ or anodic bonding^23^,^24^. The latter exploits graphitic materials provided by nature or man-made exhibiting high purity and almost no defects, which can be transformed into graphene by micromechanical exfoliation^25^, ball-milling^26^, electrochemical exfoliation^17^,^27^, liquid-phase exfoliation^28^,^29^ and reducing graphene oxide^30^. Stacking of sheets in graphite is the result of the overlap of π orbital determining van der Waals interactions. These non-covalent forces can be simply broken by applying a shearing force to either natural graphite, highly ordered pyrolitic graphite (HOPG) or single graphite crystal once dispersed in a solvent. In such a process, which is called liquid-phase exfoliation (LPE), ultrasounds spread by an ultrasonic bath form cavitation^31^ inducing a shearing force on graphite resulting in its exfoliation^32^. The solvent is therefore playing a crucial role, since the energy required to exfoliate graphene should be balanced by the solvent-graphene interaction^33^. Only very few solvents such as N-methyl-2-pyrrolidinone (NMP), N,N-dimethylformamide (DMF), benzyl benzoate and γ-butyrolactone meet this criterion. To avoid restacking, the sheets once exfoliated needs to be stabilized against re-aggregation *via* Coulomb repulsion; this can be achieved upon addition of small molecules such as sodium dodecyl sulfate accompanied by alcohols^34^, lignin molecules^35^, or porphyrins^36^,^37^. More recently, it has been reported that the use of small organic molecules acting as dispersion-stabilizing agents can promote the exfoliation of graphite when the chosen molecules have a strong affinity for the basal graphitic planes, being these interactions also stronger than those between the solvent molecules and graphene^38^,^39^,^40^,^41^. Indeed, suitably designed molecules, possessing adsorption energy on graphene larger than the adsorption of solvent-molecules, are able to form highly ordered physisorbed monolayers on the graphene surface. In this framework, it has been recently demonstrated that the addition of simple linear alkanes of different length terminated by a carboxylic-acid headgroup during the LPE process increases not only the exfoliation yield but also the percentage of graphene monolayers^42^. Nevertheless, molecule-assisted LPE in organic solvents, and in particular when the molecules contain aliphatic tails, is limited by the fact that a maximum amount of molecules added during LPE cannot exceed 25 s.a. %, which can lead to the gelation of graphite/molecule dispersions^42^. To overcome this issue, in this manuscript we extended our study to the use of molecules which at room temperature exist as a liquid, and we investigate the effect of their addition on the LPE process performed in two different commonly used solvents, i.e. NMP and *ortho*-dichlorobenzene (*o-*DCB). In particular, we focus our attention on *n*-octylbenzene (NOTBZ) as dispersion-stabilizing agent due to its total miscibility in the selected solvents and its known capacity to promote the exfoliation yield of graphene^38^. In this framework, it has been very recently demonstrated that NOTBZ molecules can be successfully exploited to promote the exfoliation of graphene in NMP. Here we explore the effect of the presence of different volume ratios of NOTBZ in either NMP or *o-*DCB during the LPE process on the quality and quantity of produced graphene flakes. We also describe a new deposition technique used for fabrication of uniform graphene films deposited from solution on flat solid surfaces. The set-up includes an integrated heating system to guarantee the fast evaporation of the solvent. More importantly, the as-obtained graphene film shows low sheet resistance and high optical transparency.

## Results

### Liquid-Phase Exfoliation

**Figure 1:**
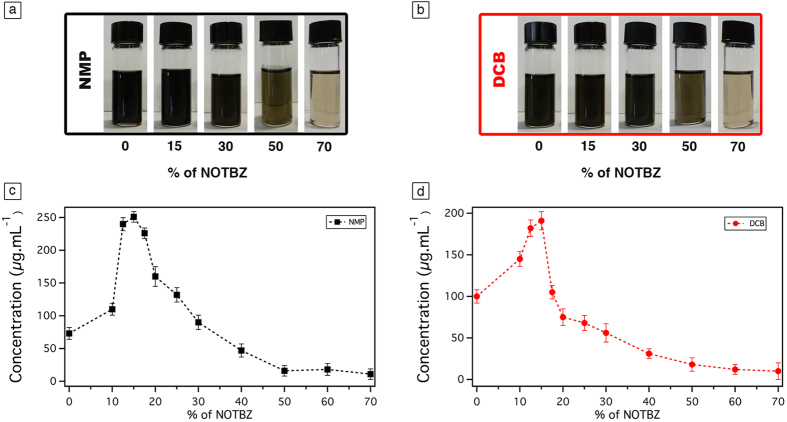
Photographs of graphene dispersions prepared by exfoliation of graphite flakes in (**a**) NMP and (**b**) o-DCB, at different percentage of NOTBZ volume. Average concentration of graphene dispersions after the filtration process at different percentage of NOTBZ volume in (**c**) NMP and (**d**) *o-*DCB. The error bars correspond to the standard deviation on the average values obtained through 10 independent experiments.

In order to cast light onto the role of the addition of NOTBZ in the graphene liquid-phase exfoliation, we first prepared dispersions by adding graphite powder in two different solvents most commonly employed for LPE, i.e. NMP and *o*-DCB (as detailed in Supplementary Information (SI), Table S1). Sonication has been applied for 6 h in an ultrasonic bath at 600 watts. The exploration was also extended to the use of 1,2,4-trichlorobenzene (TCB) and dimethylformamide (DMF) as solvents in the LPE, yet they did not give significant results (see SI-2). Different volumes of NOTBZ were added to NMP and *o-*DCB dispersions. Sonication of all samples yielded grey liquids consisting of a homogeneous phase and large numbers of macroscopic aggregates. To remove the unexfoliated graphitic material, centrifugation at 10 000 rpm for 30 min was carried out, and the obtained homogeneous dark dispersions (see Fig. 1a,b) were characterized by UV–vis–IR absorption spectroscopy (see *SI 4*). To quantify the concentration of graphene after centrifugation, graphene dispersion was mixed with chloroform (CHCl_3_) and heated up to 50 °C for 30 min and then passed through polytetrafluoroethylene (PTFE) membrane filters (pore size 100 nm). The remaining solvent and NOTBZ molecules were washed several times with diethyl ether and CHCl_3_. As previously reported^38^, the presence of adsorbed molecules on the graphene sheets may affect the mass measurements and ultimately the exfoliation yields. Careful measurements of the filtered mass provided the concentration of the dispersed phases after centrifugation.

The LPE studies started by analysing the result of two independent blank experiments performed using pure NMP and *o*-DCB. The average concentration of graphene amounted to 85 ± 5 μg mL^−1^ for NMP, and 100 ± 6 μg mL^−1^ for *o*-DCB as portrayed in Fig. 1c,d, respectively. These results are in a good agreement with those previously reported^29^,^38^ using the same experimental set-up. It is worth noting that in general the concentration of graphene in LPE process is influenced by the centrifugation speed, sonication power, temperature and time, amount and type of initial graphite, as well as the volume of the solvent.

We have then extended our LPE studies to the use of NOTBZ as dispersion-stabilizing agent. At room temperature and ambient pressure, NOTBZ is a liquid which is perfectly miscible with NMP and *o*-DCB. Among the four solvents used, upon addition of NOTBZ at specific percentage, we found that the concentration of graphene was more pronounced in NMP and *o*-DCB, and the highest yield was obtained for NMP as displayed in Fig. 1c,d. This result is in contrast with the blank experiments. Noteworthy, the most relevant increase in graphene concentration amounting to 250 μg mL^−1^ was reached upon addition to the NMP dispersion of 15 volume % of NOTBZ. Surprisingly, at this percentage, the concentration of graphene in each of the solvents even TCB and DMF (see SI Figure S1) is the highest. In the case of *o-*DCB, the maximum of graphene concentration is 190 μg mL^−1^ and is also observed upon addition of 15 volume % of NOTBZ. These concentrations correspond to an approximate increase of the exfoliation yield by 230% for NMP and 100% for *o-*DCB as compared to the blank experiments. It is worth noting that 15 volume % corresponds to a surface of graphene fully covered by NOTBZ molecules as detailed in Figure S2. Interestingly, the exfoliation at 100 volume % of NOTBZ, i.e. in pure NOTBZ, resulted in dramatic decrease of the exfoliation yield. In fact, no graphene was exfoliated, as revealed by TEM analysis. The exfoliation was successfull only when the amount of NOTBZ is below 40%. This phenomenon can be expained by the fact that NOTBZ itself is a bad solvent for liquid-phase exfoliation. While, recent reports have demonstrated that solvents with surface tensions of ca. 40 mJ m^−2^, e.g. NMP, *o*-DCB, are the best candidates for carying out LPE experiments, the relatively low surface tension of NOTBZ (ca. 30 mJ m^−2^) and notably high increase of the LPE yields in the case of NMP/NOTBZ and *o*-DCB/NOTBZ provided unambigous evidence that NOTBZ molecules can act as dispersion-stabilizing agent.

### High-Resolution Transmission Electron Microscopy (HR-TEM)

In order to characterize the exfoliated graphene sheets, qualitative analysis providing more relevant details such as the percentage of single-layer graphene (SLG) flakes, the lateral size of the flakes and the presence/absence of defects is needed. Currently, the most reliable method for the identification of the number of graphene layers in material produced by LPE is based on High-Resolution Transmission Electron Microscopy (HR-TEM). Typically, the analysis of the folded edges of the graphene sheets provides estimates of the number of layers composing the flakes. Moreover, from the TEM analysis, the average lateral size of the graphene sheets can be defined.

**Figure 2:**
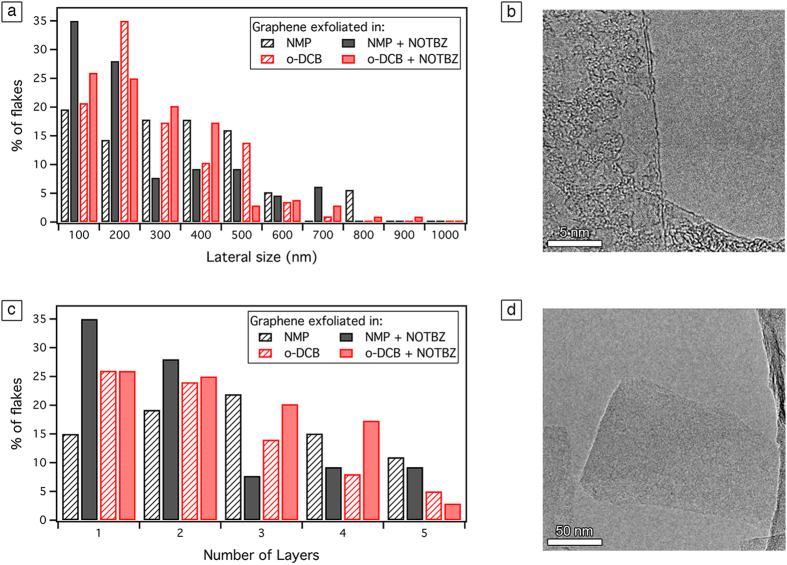
Statistical analyses of HR-TEM results of graphene exfoliated in pure NMP and o-DCB in the presence of NOTBZ molecules. (**a**) Histogram of the lateral flake size distribution, (**b**) HRTEM micrograph of a monolayer, (**c**) histogram of the distribution of the flake thickness, and d) TEM micrograph of a monolayer.

First, we discuss dispersions prepared in NMP in the absence and/or the presence of NOTBZ molecules at the highest concentration obtained, i.e. the 15%. The TEM micrographs reveal that the majority of dispersions in NMP are composed of a large fraction of monolayer 15% and bilayer 18% graphene sheets folded over themselves with lateral size smaller then 500 nm. In the presence of NOTBZ, graphene flakes were found mainly in a monolayer 35% and in a bilayer 28% form (Fig. 2c). Also, the majority of flakes were found to be smaller than 200 nm (Fig. 2a). Figure 2b,d show HRTEM images of monolayered graphene.

We then focused our attention on the samples prepared in *o-*DCB in the presence and/or absence of NOTBZ as dispersion-stabilizing agent at the highest concentration obtained, i.e. the 15%. The TEM micrographs reveal that the majority of dispersions in *o-*DCB are composed of a large fraction of monolayer 25% and bilayer 22% graphene sheets folded over themselves with lateral size smaller then 500 nm (Fig. 2a). In the presence of NOTBZ, 25% of graphene flakes were found mainly in a monolayered form and 24% in a bilayered one. Additionnaly, the majority of flakes were found to be smaller than 500 nm.

### Raman Spectroscopy

**Figure 3:**
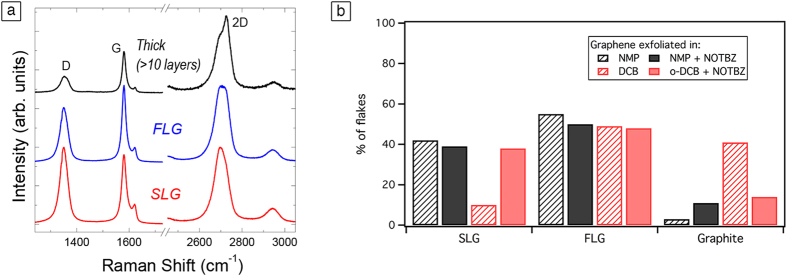
(**a**) Typical Raman spectra observed for dispersions obtained by adding NOTBZ. (**b**) Statistical analysis, based on Raman spectroscopy, on the thickness distribution of the flakes in NMP and DCB before and after addition of NOTBZ.

To complement the characterization result by HR-TEM, Raman spectroscopy was used. In fact, Raman analysis is more qualitative and it has been shown that this technique is sensitive to the changes in thickness composition under different experimental conditions. It has been revealed that Raman spectroscopy offers a simple and fast analysis of graphene sheet thickness with the evolution of 2D peak shape^43^,^44^. However, due to the effect of the solvent, edge effects and strain and doping caused during exfoliation, the Raman spectrum of LPE graphene is more difficult to analyze compared to the spectrum of mechanically exfoliated graphene. Therefore, the analysis is only quantitative and must be used in combination with HRTEM results. Figure 3a shows the typical Raman spectra of LPE graphene obtained by adding NOTBZ to different solvents. Based on the shape of the 2D peak, we can distinguish between single-layer (SLG), few-layers graphene (FLG) and graphite (>10 layers). Statistical analysis shows that upon addition of NOTBZ to NMP and *o-*DCB, the percentage of SLG strongly increases for *o-*DCB, while it stays constant for NMP (Fig. 3b). Therefore, based on Raman spectroscopy, NOTBZ seems to have a bigger effect on *o-*DCB than NMP. Figure 3 shows that the increase observed of single-layer concentration observed for *o-*DCB is related to a decrease in the percentage of thick layers, showing that NOTBZ is an effective exfoliation agent in *o-*DCB. Note that all the spectra show a D peak, which is typical for LPE graphene^29^. We did not observe any particular difference in the intensity ratio between the D peak and G peak, I(D)/I(G), which indicates that NOTBZ does not introduce any structural defect in graphene when added either in NMP or *o-*DCB (see SI, Table S2).

Noteworthy, the thickness analysis based on Raman spectroscopy is only quantitative and must be used in combination with HR-TEM results. Commonly, the results based on TEM are more reliable for liquid-phase exfoliated graphene, and the determination of number of layers based on Raman spectra is more suitable for graphene prepared by micromechanical exfoliation and CVD methods. Therefore, the small discrapancy in the flake thicknes analysis between the Raman and TEM is not surprising.

### Graphene thin film

**Figure 4:**
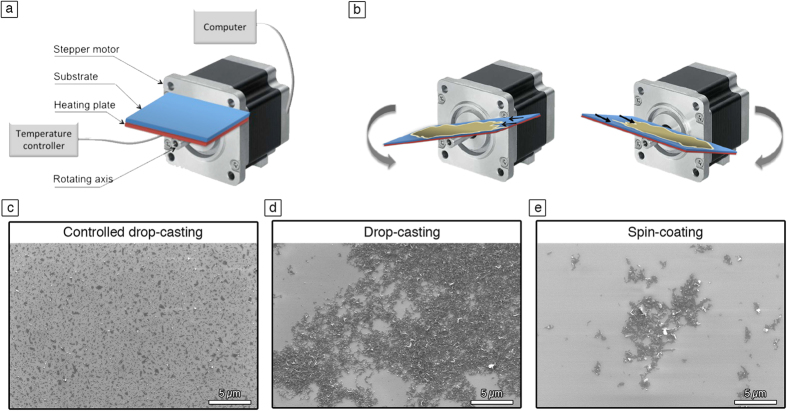
(**a**) Schematic scheme of the developed technique for depositing graphene dispersions. (**b**) Operating principle of the technique showing a rotation of the axis in a counterclockwise direction (image on the left) and a clockwise direction (image on the right). SEM images of graphene film prepared by (**c**) spin-controlled drop-casting (using the set-up in (**a**,**d**) drop-casting, and (**e**) spin-coating.

Despite the increase of the exfoliation yield and the successful production of mono and few layers thick graphene sheets, the deposition of these graphene dispersions on commonly used substrates (mainly SiO_2_ and quartz) to form uniform films for applications in electronics remains a challenge. The solvents used for LPE have high boiling points, which results in a re-aggregation problem particularly when drop-casting method is used. Moreover, spin-coating of such dispersions is very challenging because as a results of the poor wetting, only few graphene sheets remains on the surface of the substrate, making it an unsuitable technique to form uniform films of graphene. Another well-known phenomenon observed when a drop of graphene dispersion is drying on a surface, is the coffee-ring effect^45^,^46^,^47^. Some graphene flakes accumulate at the drop periphery, yielding a characteristic ring shape pattern. Consequently, we thought of controlling the deposition from solution by devising a new simple technique, called spin-controlled drop-casting. It consists of a stepper motor featuring a rotating axis controlled by a custom software as shown in Fig. 4a. The substrate is connected to a heating plate fixed on the axis. First the drop is deposited at a zero angle, i.e. when the substrate is in its planar configuration. Then the x-axis is rotated back and forth in counterclockwise (Fig. 4b left) and clockwise directions (Fig. 4b right) by the stepper motor at controlled speed and angles configured by the software. These movements accompanied by the underneath heating of the substrate allow to avoid the coffee ring-effect and obtain a complete evaporation of the solvent. Interestingly, a uniform film with more dispersed and less aggregated sheets is obtained (see Fig. 4c) unlike the normal casting method (see Fig. 4d) or spin coating (see Fig. 4e). Moreover, this new method allows the fabrication of graphene films fully covering the substrate on a scale of 100 μm^2^ (see Figure S13). AFM and SEM were used in order to investigate the change of morphology of these films, when different numbers of deposition (*n*) are applied. The graphene sheets are forming islands (as observed in Fig. 4c) with a height up to 24 ± 4 nm (Figure S14). The subsequent depositions of multiple dispersion drops make it possible to increase the surface coverage of the film, while the thickness of the films remains constant at 24 nm. Noteworthy, as many as nine depositions (*n* = 9) is needed in order to form a film with more than 95% of surface coverage. A correlation between the coverage and the number of deposition has been extracted from AFM analysis (Figure S15).

**Figure 5:**
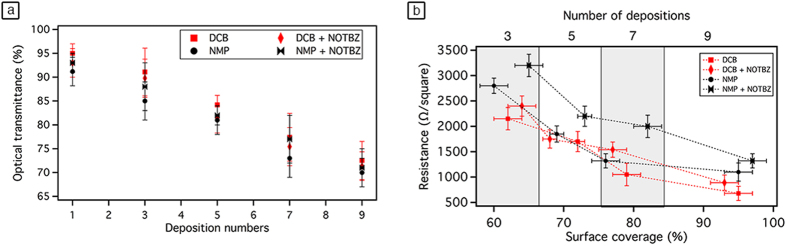
(**a**) The optical transmittance at λ = 550 nm, and (**b**) the sheet resistance of graphene film versus number of deposition. The error bars correspond to the standard deviation on the average values obtained by performing 6 independent measurements.

Optical transmittance and electrical resistance of graphene films are two important characteristics for many opto-electronic applications requiring transparent electrodes. Therefore, benefiting of the advantages offered by our developed set-up, we prepared on quartz substrates conductive graphene films from graphene dispersions in *o*-DCB or NMP in the presence and absence of NOTBZ molecules. The preparation mechanism is depicted in Fig. 4. Prior to deposed graphene dispersions, quartz substrate were ozone treated in order to clean the surface. UV–vis spectroscopy was used to measure the optical transmittance versus the number of deposition as shown in Fig. 5a. In the visible range, graphene films prepared in *o*-DCB or NMP, with and without NOTBZ molecules, show transmittance of ~93% at 550 nm for the first deposition (*n* = 1) regardless the type of solvent or the presence or absence of NOTBZ. In all cases and as expected, the transmittance decreases when the number of deposition or the surface coverage increases. For *n* = 9, where the sample is uniform with coverage exceeding 95%, the transmittance at 550 nm is lowered as compared to a single deposition and it decreases from ~93 to 72%.

The measured sheet resistances of the 24 nm thick graphene film led to very high values for films prepared from only three depositions (see Fig. 5b). This is mainly due to the fact that these sheets are not perfectly overlapped. Average resistance values were in the range of 2500 to 1000 Ω/square for graphene prepared from *o*-DCB, and were higher in the case of NMP (3200 to 2200 Ω/square) and in the presence of NOTBZ because of the low conductivity of the molecule. However, if more depositions are performed, the film resistance decreases (see Fig. 5b) due to the better physical interconnection among adjacent graphene sheets and the increased surface coverage. The presence of the added molecules, because of their electrically insulating nature, results in higher resistances as well. Our best sample (graphene from *o*-DCB only) shows a surface coverage close to 100% for 9 depositions with 72% of transmittance and 680 Ω/square. With a surface coverage amounting 100%, the thickness of the graphene film times the sheet resistance value expressed in Ω/square is equal to the volume resistivity in Ω m of the graphene film. In our case the volume resistivity is as low as 1.6 × 10^−5^ Ω m. Also the sheet resistances and transparency reported in this manuscript are very close to those of graphene deposed by casting-dropping graphene/acetone suspension onto a polystyrene glass ride (~1000 Ω/square, 80% at 550 nm)^48^, or electro-chemically exfoliated graphene treated with HNO_3_ (~400 Ω/square and 73% at 550 nm)^49^ but still far from CVD-graphene doped with HNO_3_ (~30 Ω/square and 90% at 550 nm)^50^.

## Discussion

We have demonstrated that it is possible to enhance the yield of graphite exfoliation by mastering a molecule-assisted LPE-based approach to produce high-quality graphene flakes from bulk graphite. To attain a fundamental understanding of the role of molecule-graphene interaction on the yield of graphene LPE, we have carried out a comparative study by using NOTBZ as dispersion-stabilizing agent upon increasing the volume ratio of NOTBZ in two organic solvents, i.e. NMP and *o-*DCB. Careful analysis revealed a significant increase in the yield of exfoliation with the volume percentage of NOTBZ until a maximum. In particular, the most effective exfoliation was obtained with 15 volume % of NOTBZ in NMP as a solvent, with a concentration of exfoliated graphene of 250 μg mL^−1^ corresponding nearly to 230% increase in exfoliation yield when compared to samples prepared in pure NMP (85 μg mL^−1^). In the case of o-DCB, the most effective exfoliation was observed using also 15 volume % of NOTBZ, which led to ca. 100% increase in the exfoliation yield. Furthermore, a remarkable increase of single-layer graphene (SLG) flakes was observed in some cases. Careful investigation made it possible to correlate the 15% NOTBZ volume to 100% of surface area estimated which was found to be accurate for all studied solvents. TEM analysis of dispersions produced in NMP revealed that the quantity of SLG is improved by 100% when using NOTBZ molecules. In the case of *o-*DCB, the quantity of SLG was found amounting 25% even after addition of NOTBZ molecules, although a qualitative Raman analysis showed an opposite trend between NMP and *o-*DCB after adding NOTBZ. Graphene ink produced with our approach was deposed uniformly on SiO_2_ or quartz substrate by using a new deposition technique relying on a spin-controlled drop casting. These graphene films exhibit sheet resistance as low as 1 kΩ/square and transparency of 70% at 550 nm. By controlling the number of depositions and the concentration of graphene dispersions, film resistance and transparency can be tuned. Our approach opens up new avenues for the technological applications of graphene ink as low-cost electrodes and conducting nanocomposite for electronics.

## Methods

### Materials

N-methyl-2-pyrrolidinone (NMP - product number *p.n.* 332461) and *ortho*-dichlorobenzene (*o-*DCB, *p.n.* 240664) were purchased from Sigma-Aldrich and used as solvent for the exfoliation. *N*-octylbenzene (NOTBZ, *p.n.* 113190) and graphite powder (*p.n.* 332461) were also acquired from Sigma-Aldrich and used without further treatment.

### Preparation of graphene by exfoliation of graphite

Graphite flakes were sonicated for 6 h at 40 ± 2 °C (600 W) in two different solvents, i.e. NMP and *o-*DCB. Since NOTBZ is a liquid at room temperature, we decided to fix the total volume of the mixture at 10 mL. For each solvent, we varied the amount of the added molecules and fix the amount of graphite powder. The latter was changed for each of the solvents used (see Supplementary Information SI for details). Sonication of graphite powder led to grey liquid consisting of a homogeneous phase and large numbers of macroscopic aggregates. As previously reported^29^, these aggregates can be removed by centrifugation (Eppendorf 5804, rotor F-34-6-38, 30 min at 10 000 rpm), yielding to a homogeneous dark dispersion.

### Characterization

The amount of exfoliated graphene was measured on a Cubis Ultramicro-Balance MSA2.7S-000DM using a membrane holder. The filters were weighed 5 times before and after the filtration process in order to average the mass of graphene. Dispersions were first transferred in 10 mm path length quartz cuvette and analyzed by means of UV-vis-IR absorption spectroscopy using a Jasco V670 spectrophotometer equipped with a Peltier thermostated cell holder at 20.00 ± 0.05 °C. Also the optical transmittance of graphene films was measured using the same spectrophotometer equipped with a transmittance holder. The number of layers of graphene flakes was determined by High-Resolution Transmission Electron Microscopy (HR-TEM) using a JEOL 2100 F microscope. TEM samples were prepared by drop-casting the graphene dispersions on standard Quantifoil® R 2/1 holey carbon copper grid, followed by solvent evaporation at 150 °C for 10 min. Micro Raman measurements were carried out with a Renishaw spectrometer in confocal mode and backscattering geometry with excitation energy of 488 nm, 514.5 nm and 633 nm. A 100X objective with a NA of 0.95 was used for the characterization. This ensured a small laser spot size of less than 400 nm. The spectral resolution of the spectrometer is 2–3 cm^−1^. The laser power was well below 1 mW to avoid damage. Around 50 flakes drop-cast on silicon substrates were analyzed and the shape of the 2D peak and intensity (as height) ratio between D and G peak, I(D)/I(G) were analyzed. In particular, the 2D peak was fitted with a Lorentzian lineshape and the coefficient of determination, R^2^, was used to discriminate between monolayer (symmetric peak), few-layers graphene (asymmetric 2D peak) and graphitic (2 peaks). Such an approach allowed us to gain qualitative information on the number of layers distribution and the amount of defects. Atomic Force Microscopy (AFM) characterization was carried out by using a Veeco Dimension 3100, running with a Nanoscope IV controller. The analysis of AFM images and the extraction of the topographical profiles, for measuring the thickness of the different films, were done using Gwyddion.

### Graphene thin film and sheet resistance measurements

Prior to use, quartz substrates were washed by applying 2 cycles of acetone and isopropanol and cleaned by UV ozone treatment. 70 μL of the as-prepared graphene dispersions in *o*-DCB or NMP were deposed on freshly cleaned quartz substrate using our spin-controlled drop-casting set-up. A custom high torque step motor, with 1.8°/step and 200 step/revolution was used. We explored different parameters, i.e. rotation angle (from 0 to 35 steps), speed (from 1 to 35 step/s) and temperature (from 20 to 80 °C). The best condition giving a homogeneous film was by setting the rotating angle and speed at 22 steps and 11 steps/s respectively while heating at 50 °C. One deposition corresponds to one cycle of drop-casting 70 μL of graphene dispersion, 30 minutes of rotation and 180 min of post annealing at 100 °C under vacuum. The numbers of deposition were varied between 1 and 9. Sheet resistances were measured with a four probes Jandel (RM3000) platform.

## Additional Information

**How to cite this article**: Haar, S. *et al*. Enhancing the Liquid-Phase Exfoliation of Graphene in Organic Solvents upon Addition of *n*-Octylbenzene. *Sci. Rep.* **5**, 16684; doi: 10.1038/srep16684 (2015).

## Supplementary Material

Supplementary Information
