# Supplementary material for: Enhancing the Liquid-Phase Exfoliation of Graphene in Organic Solvents upon Addition of n-Octylbenzene
Source: Sci Rep. 2015 Nov 17;5:16684. doi: 10.1038/srep16684 (PMC4648096; doi:10.1038/srep16684)
Supplement: Supplementary Information [file srep16684-s1.pdf]

Supplementary Information

Enhancing the Liquid-Phase Exfoliation of Graphene in Organic Solvents upon Addition of *n*-Octylbenzene.

Sébastien Haar[1], Mirella El Gemayel[1], Yuyoung Shin[2], Georgian Melinte[3], Marco A. Squillaci[1], Ovidiu Ersen[3], Cinzia Casiraghi[2], Artur Ciesielski[1,*] and Paolo Samorì[1,*]

[1] ISIS & icFRC, Université de Strasbourg & CNRS, 8 allée Gaspard Monge, 67000 Strasbourg, France.

[2] School of Chemistry, Oxford Road, University of Manchester, Manchester M13 9PL, United Kingdom.

[3] Institut de Physique et Chimie des Matériaux de Strasbourg (IPCMS), UMR 7504, 23 rue du Loess, 67037 Cédex 08 Strasbourg, France.

* Corresponding author: samori@unistra.fr , ciesielski@unistra.fr

Table of content

1. Materials and methods S-2

2. Graphene concentration in different solvents S-3

3. Surface area S-4

4. UV-Vis-IR spectroscopy S-5

4.1. Exfoliation in NMP in the presence/absence of NOTBZ S-5

4.2. Exfoliation in o-DCB in the presence/absence of NOTBZ S-6

4.3. Exfoliation in TCB in the presence/absence of NOTBZ S-6

4.4. Exfoliation in DMF in the presence/absence of NOTBZ S-7

4.5. Lambert Beer behavior S-7

5. Stability tests S-8

6. X-ray photoelectron characterization (XPS) S-9

7. HR-TEM S-10

8. Raman S-12

9. New deposition set-up S-13

1. Materials and methods

In order to obtain graphene dispersions, graphite flakes were sonicated for 6 h at 40 ± 2°C (600 W) in the chosen solvents, keeping the total reaction volume constant, i.e. 10 mL. Since the ratio between graphite powder and the solvent was kept constant, i.e. 1 wt %, different masses of graphite flakes were used, and details have been reported in table S1.

Volume percentage	*Volume NOTBZ (mL)*	*Volume solvents (mL)*	*Final volume (mL)*	*Mass graphite (mg)*			
				DMF	NMP	DCB	TCB
0 %	0	10	10	92	100	126	150
10 %	1	9	10	92	100	126	150
12.5 %	1.25	8.75	10	92	100	126	150
15 %	1.5	8.5	10	92	100	126	150
17.5 %	1.75	8.25	10	92	100	126	150
20 %	2	8	10	92	100	126	150
25 %	2.5	7.5	10	92	100	126	150
30 %	3	7	10	92	100	126	150
40 %	4	6	10	92	100	126	150
50 %	5	5	10	92	100	126	150
60 %	6	4	10	92	100	126	150
70 %	7	3	10	92	100	126	150
80 %	8	2	10	92	100	126	150
90 %	9	1	10	92	100	126	150
100 %	10	0	10	92	100	126	150

Table S1. Masses of graphite, solvent and organic molecules used in LPE process.

2. Concentration of graphene in the different solvents

To quantify the concentration of graphene after centrifugation, a mixture of graphene dispersion and chloroform was first heated up to 50 °C for 30 minutes and then passed through polytetrafluoroethylene membrane filters (pore size 100 nm). The remaining solvent and NOTBZ molecules were washed several times with diethyl ether and chloroform and dried for 1 day in a vacuum oven at 50 °C. Careful measurements of the filtered mass were performed on a microbalance (Sartorius MSA2.75) to give the concentrations of graphene. The presence of adsorbed molecules between sheets may affect the mass measurements and ultimately the exfoliation yields. Thus, heating was necessary to completely remove the molecules from graphene. Exfoliation of graphite in four solvents in the presence of NOTBZ leads to different concentrations that are depending of the NOTBZ volume percentage. Surprisingly, 15 % give the largest concentration for all the solvents. Although the increase is not as impressive compared to NMP and *o*-DCB, DMF and TCB exhibit their higher concentration at the same ratio.

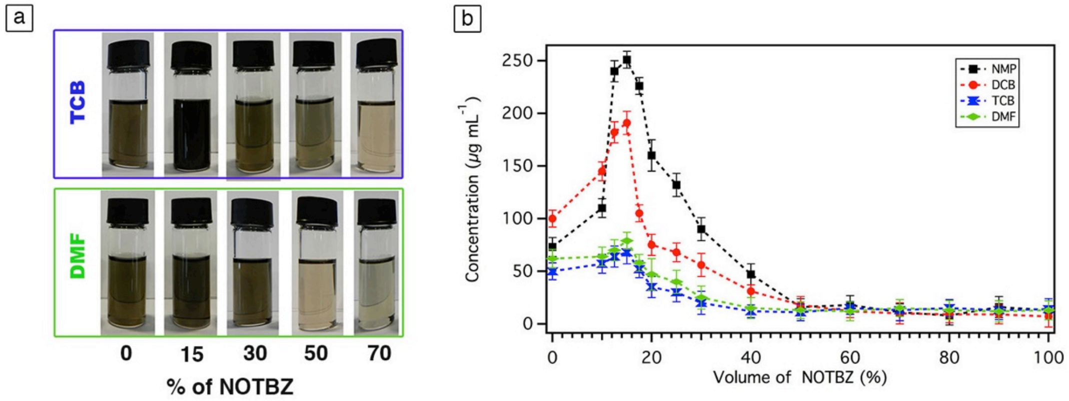

Figure S1. a) Photographs of graphene dispersions prepared by exfoliation of graphite flakes in TCB and DMF at different NOTBZ volume percentage, b) average concentration of graphene dispersions after the filtration process. The error bars correspond to the standard deviation on the average values obtained through 10 independent experiments.

3. Surface area

Typically NOTBZ does not form any ordered monolayers at the HOPG/graphene surface at room temperature and ambient pressure. Nevertheless, theoretical assumption of NOTBZ packing motif on graphene has been used for calculation the coverage areas. The area occupied by single molecule amounts to A= (5.92± 0.9) nm^2. The areas A occupied by single dispersion-stabilizing molecules, as well as area of graphene unit cell ($G_{unit\ cell}$ = (0.052± 0.004) nm^2) estimated by theoretical assumption can be used for calculating the mass and number of dispersion-stabilizing molecules needed to cover accessible graphene area. Noteworthy, in our calculations graphite powder has been considered as a single (rectangular) graphene sheet.

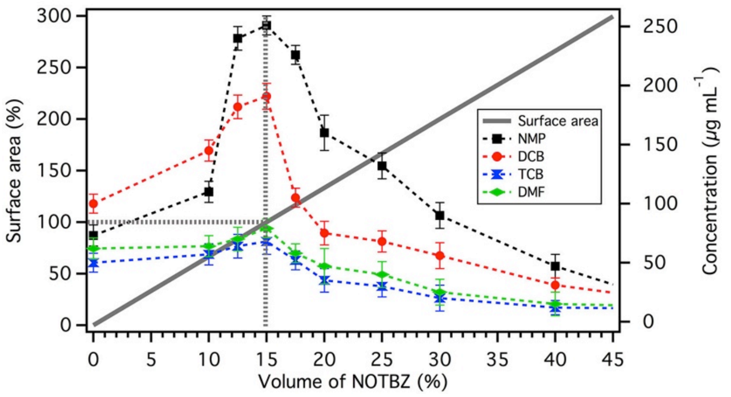

Figure S2. Correlation between surface area and volume percentage for all dispersions.

We found a correlation between the best ratio, i.e. 15 % and the surface area coverage. Interestingly, only 100 % of graphene coverage gives the highest exfoliation yield and for all the solvents we tested.

4. UV-Vis-IR spectroscopy

All dispersions were also characterized by UV–vis–IR absorption spectroscopy. The spectra are as expected being featureless in the visible – IR region. Each of these dispersions was diluted a number of times and the absorption spectra recorded. The absorbance (660 nm) divided by cell length is plotted versus concentration. A Lambert – Beer behavior was observed for all samples.

4.1 Graphene exfoliated in NMP in the presence/absence of NOTBZ with the highest concentration.

Graphene dispersions were characterized by UV–vis–IR absorption spectroscopy. As expected, the spectra are featureless in the visible – IR region. Each of these dispersions in NMP, i.e. graphene, graphene + NOTBZ was diluted a number of times and the absorption spectra recorded.

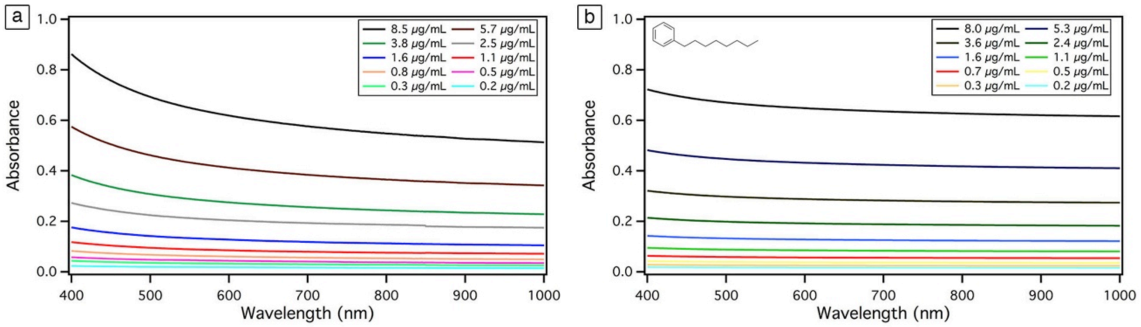

Figure S3. UV–Vis spectra of graphene dispersion in NMP in the presence/absence of NOTBZ at the highest concentration.

4.2 Graphene exfoliated in *o*-DCB in the presence/absence of NOTBZ with the highest concentration.

Graphene dispersions were characterized by UV–vis–IR absorption spectroscopy. As expected, the spectra are featureless in the visible – IR region. Each of these dispersions in *o*-DCB, i.e. graphene, graphene + NOTBZ was diluted a number of times and the absorption spectra recorded.

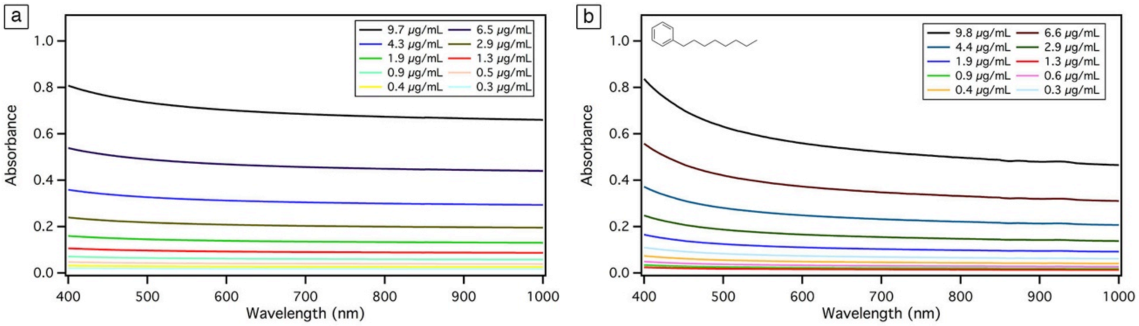

Figure S4. UV–Vis spectra of graphene dispersion in *o*-DCB in the presence/absence of NOTBZ at the highest concentration.

4.3 Graphene exfoliated in DMF in the presence/absence of NOTBZ with the highest concentration.

Graphene dispersions were characterized by UV–vis–IR absorption spectroscopy. As expected, the spectra are featureless in the visible – IR region. Each of these dispersions in DMF, i.e. graphene, graphene + NOTBZ was diluted a number of times and the absorption spectra recorded.

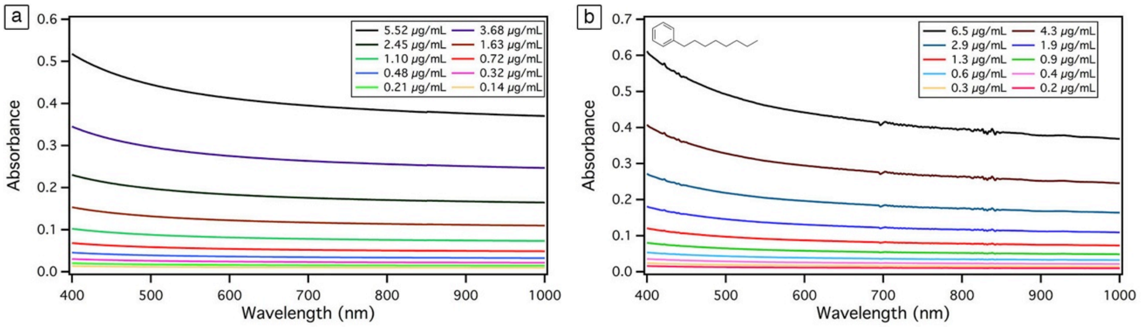

Figure S5. UV–Vis spectra of graphene dispersion in DMF in the presence/absence of NOTBZ at the highest concentration.

4.4 Graphene exfoliated in TCB in the presence/absence of NOTBZ with the highest concentration.

Graphene dispersions were characterized by UV–vis–IR absorption spectroscopy. As expected, the spectra are featureless in the visible – IR region. Each of these dispersions in TCB, i.e. graphene, graphene + NOTBZ was diluted a number of times and the absorption spectra recorded.

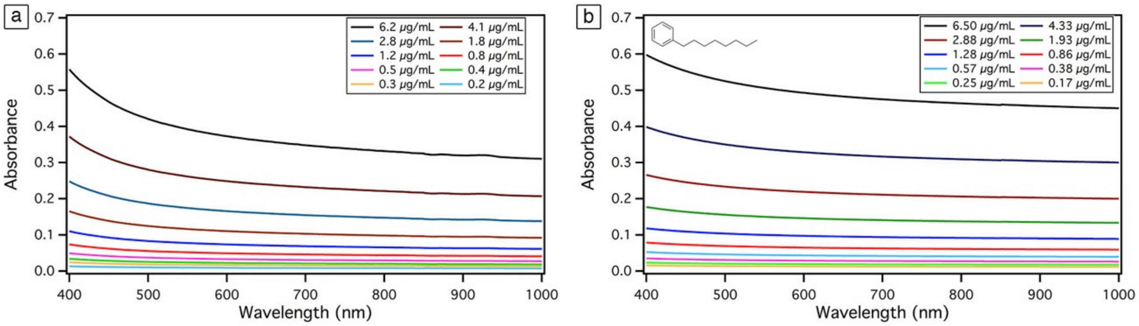

Figure S6. UV–Vis spectra of graphene dispersion in TCB in the presence/absence of NOTBZ at the highest concentration.

4.5 Lambert Beer behaviors

The absorbance at 660 nm divided by cell length is plotted versus concentration. A Lambert – Beer behavior was observed for all samples, with extracted values of the regression from linear fitting amounting 0.98 for all samples.

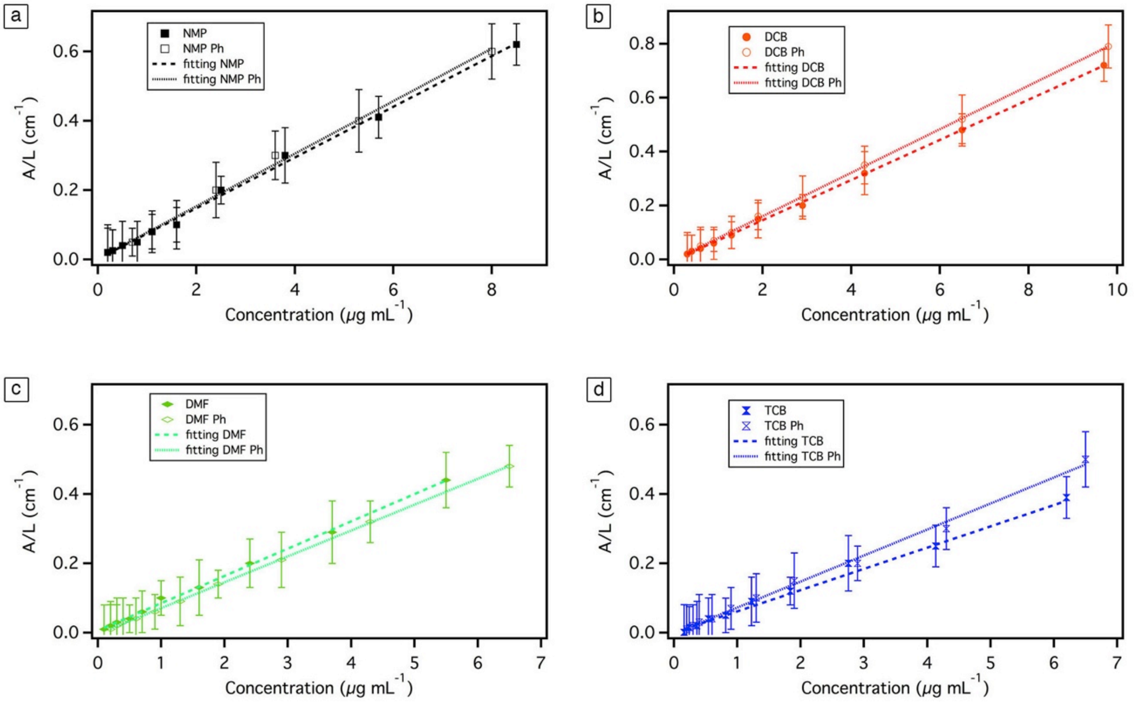

Figure S7. Optical absorbance (λ= 660 nm) divided by cell length (A/L) as a function of concentration showing Lambert-Beer behavior of all dispersions.

5. Stability tests

We studied the stability of graphene dispersions in the presence/absence of NOTBZ molecules during 1 year. NMP is showing stability over 1 year in the presence of NOTBZ. Also *o*-DCB, TCB and DMF dispersions show better stability in the presence of NOTBZ.

	NMP		DCB		TCB		DMF	
Months	Ø	NOTBZ	Ø	NOTBZ	Ø	NOTBZ	Ø	NOTBZ
1	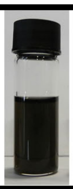	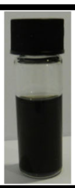	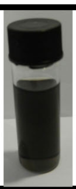	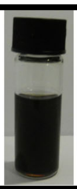	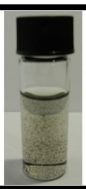	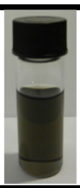	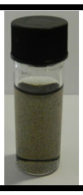	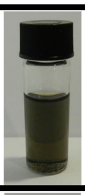
6	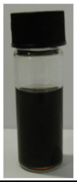	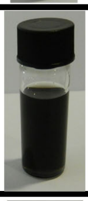	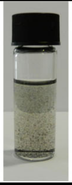	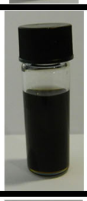		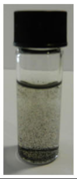		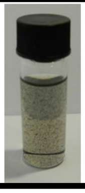
12	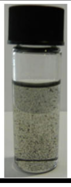	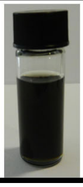		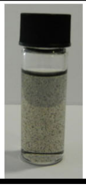				

Figure S8. Photographs of graphene dispersions.

6. X-ray photoelectron characterization (XPS)

XPS analyses were carried out on a Thermo Scientific K-alpha X-ray photoelectron spectrometer with a basic chamber pressure of ~10^{-8} mbar and Al anode as the X-ray source (x-ray radiation of 1486 eV). Spot sizes of 400 µm were used and pass energies of 200 eV for survey scans and 50.00 eV for high-resolutions scans were used. 150 µL of dispersions were spin coated on Au substrate for 1 minutes at 1000 rpm and substrates were annealed for 1 day at 100 °C in a oven under vacuum.

We used XPS to analyze graphene + NOTBZ in NMP before and after washing the NOTBZ. The high-resolution C1s XPS spectrum in Fig. S9a of the graphene sheets from NMP dispersions showed a sharp peak at 284.3 eV that corresponded to C-C bonds of carbon atoms in a conjugated honeycomb lattice. Figure S9b represents the high-resolution C1s XPS spectrum of graphene + NOTBZ showed a larger peak at 284.6 eV corresponding to delocalized π conjugation from the sp^2 atomic structure of graphite. Also this peak exhibits a larger behavior as the C-C bond from NOTBZ is also contributed. After the washing step, the peak at 284.4 eV became sharp again (Figure S9c) and confirmed the removal of NOTBZ.

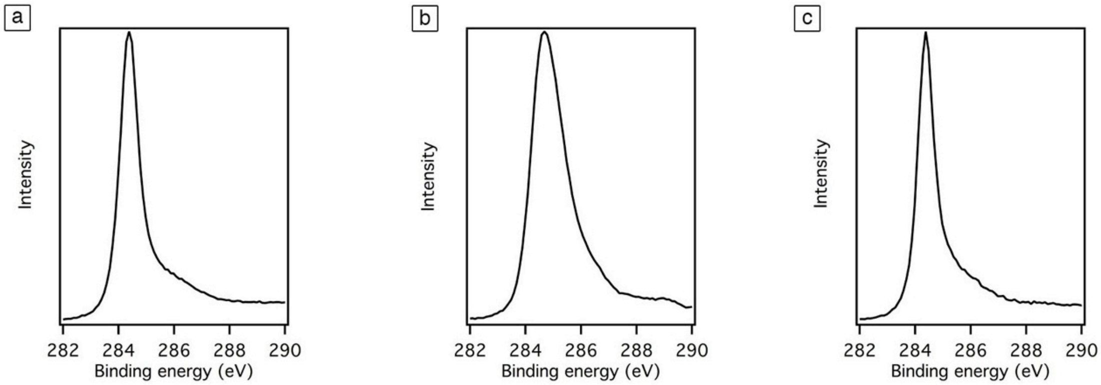

Figure S9. High-resolution C1s spectra of a) graphene exfoliated in NMP, b) graphene exfoliated in the presence of NOTBZ in NMP and c) graphene exfoliated in the presence of NOTBZ in NMP after washing the NOTBZ molecules.

7. HR-TEM

All graphene dispersions were also investigated using High Resolution Transmission Electron Microscopy (HR-TEM). For the analyses, a drop of solution containing graphene flakes was deposited on a TEM grid covered by a lacey carbon membrane. The as-prepared grids were kept overnight under vacuum for accelerating the solvent evaporation. The experimental data were recorded on a JEOL 2100 F working at 200kV, equipped with a Cs probe corrector and a GATAN Tridiem imaging filter. To increase the graphene stability at high magnification, between each recorded image, the electron beam was electronically moved from the sample surface allowing thus the electron discharge. In this way up to 5 images can be acquired before damaging the sample. In a number of cases we observed folded monolayer graphene sheets as shown in Fig. S9a. The edges of graphene with different number of layers are also shown in Fig. S9.

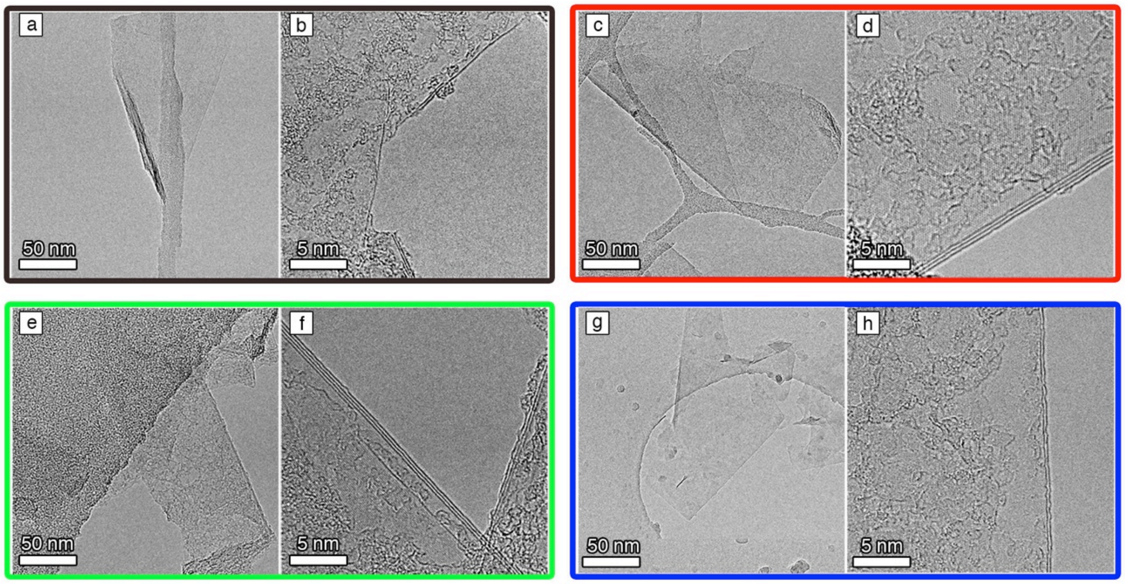

Figure S10. a), c), e) and g) TEM micrographs of mono-bilayer graphene flakes prepared in NMP, *o*-DCB, DMF and TCB. b), d), f) and h) HR-TEM micrographs of graphene flakes prepared in NMP, *o*-DCB, DMF and TCB.

By analyzing hundreds of TEM micrographs, we were able to make statistic on the number of layers and the size of graphene sheets as shown in Fig. S11. Exfoliation in

the presence of NOTBZ in the four solvents leaded to a majority of mono and bi-layer flakes with more than 50 % of the total graphene flakes. More than 25 % of the total flakes are present in a monolayer form. But the size of the exfoliated graphene sheets is comprised between 100 and 400 nm. For DMF and TCB, the majority of graphene flakes have a lateral size larger than 200 nm.

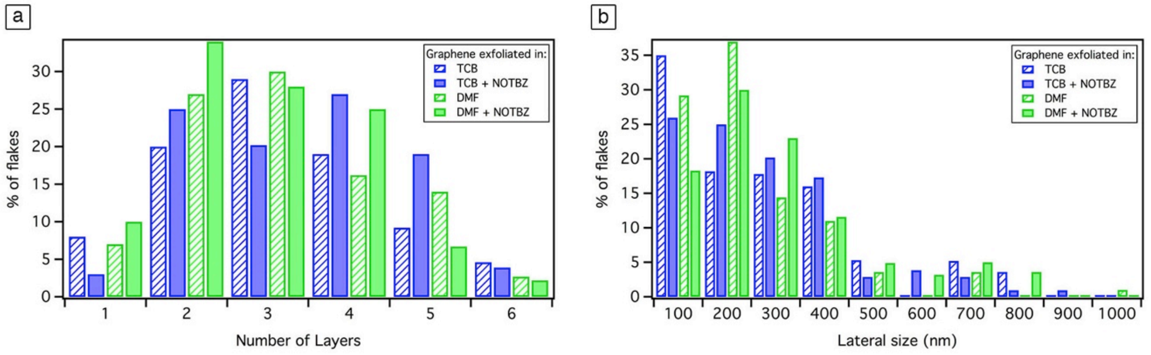

Figure S11. Statistical analyses of TEM and HR-TEM results of graphene dispersions exfoliated in the presence of NOTBZ in NMP, o-DCB, DMF and TCB. a) Histograms of the number of layer distribution of graphene flakes and b) histograms of the lateral size flakes size distribution of graphene flakes.

8. Raman

The first order Raman spectrum is characterized by G and D peak. The D peak is typically observed in graphene obtained by LPE and it is attributed to finite size (i.e. D peak activated by the edges). Therefore, its intensity depends on the size of the flakes (and also on the excitation energy). Table S2 shows the results obtained on thickness distribution in different solvents and I(D)/I(G) obtained from the Raman spectra. Figure S11 shows the thickness distribution in Table 1. Raman spectroscopy shows that NOTBZ is an effective agent for exfoliation of graphite in o-DCB, while it seems to be less efficient in NMP and TCB. In the case of DMF, the addition of NOTBZ results in a strong decrease of the single-layer content in the dispersion. Those data are not in agreement with TEM. However, one must remember that Raman spectroscopy has been performed with an optical microscope on flakes deposited by drop-casting on a silicon substrate. Therefore, small size flakes (<300 nm) may be difficult to spot, in contrast to TEM analysis, which mainly focuses on small size flakes.

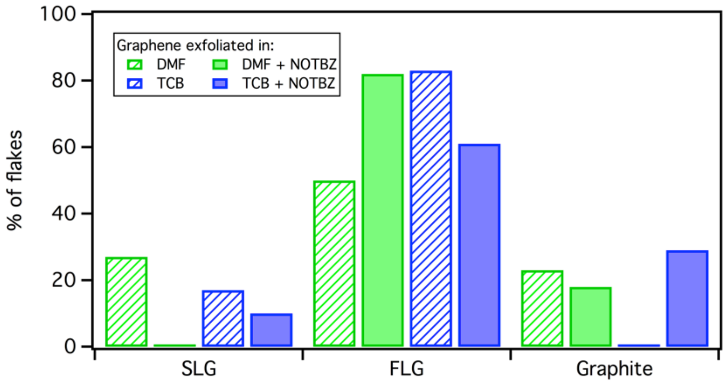

Figure S11 Statistical analysis, based on Raman spectroscopy, on the thickness distribution of the flakes in NMP and *o*-DCB before and after adding NOTBZ.

	% of flakes	I(D)/I(G)	% of flakes	I(D)/I(G)	% of flakes	I(D)/I(G)
	SLG		FLG		Graphite	
**NMP+NOTBZ**	38.9	0.6-1.9	50	0.3-1.8	11.1	0.3-0.6
***o*-DCB+NOTBZ**	38	0.6-1.7	48	0.2-1.3	14	0.2-0.7
**DMF+NOTBZ**	0	NA	82	0.3-1.2	18.4	0.2-0.5
**TCB+NOTBZ**	10.2	0.6-0.7	61	0.3-0.9	28.6	0.2-0.4

Table S2 Comparative table of the % of flakes and ratio I(D)/I(G) for the four solvents in the presence of NOTBZ molecules.

9. New deposition set-up description

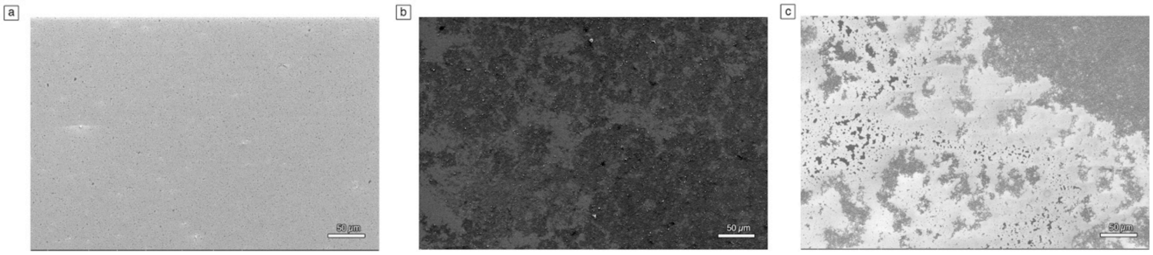

Figure S13. Large-scale SEM images of graphene film for N=1 prepared by a) spin-controlled drop-casting, b) drop-casting, and c) spin-coating.

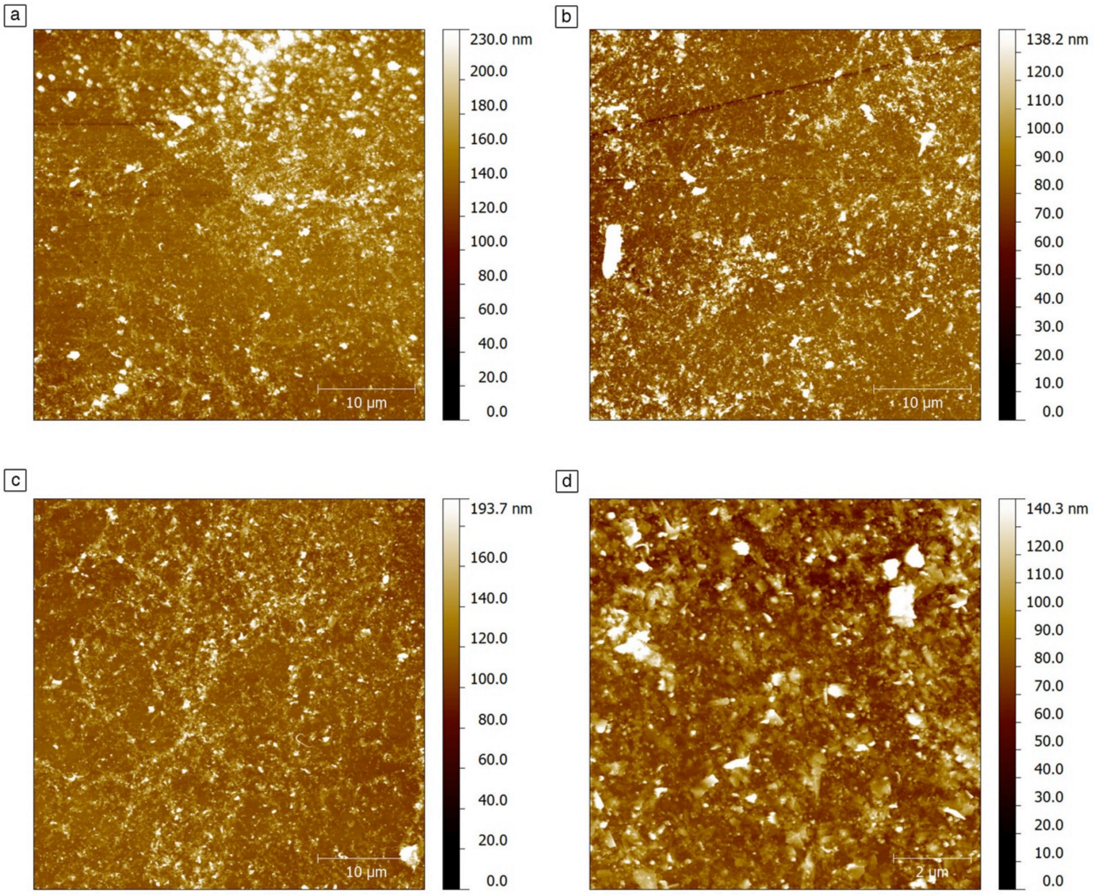

Figure S14 AFM images of graphene film prepared by spin-controlled drop-casting for a) N=3, b) N=5, c) N=7 and d) N=9 from NMP + NOTBZ dispersion.

Surface coverage was estimated by analyzing AFM images for each deposition. In particular, the average surface coverage was measured using Gwyddion's statistical analysis from different images with different scan sizes.

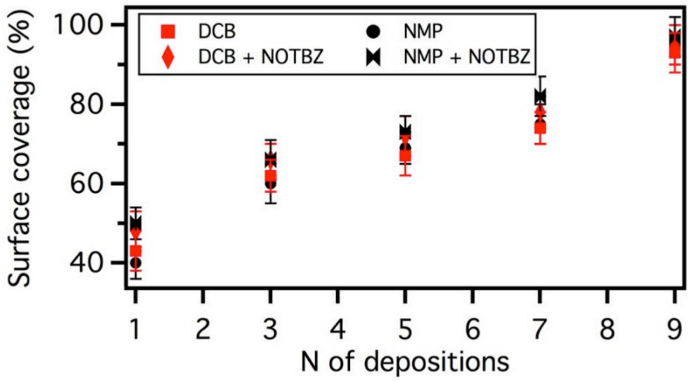

Figure S15 Estimation of the surface coverage.
